# Advances and Challenges in Biomarkers Use for Coronary Microvascular Dysfunction: From Bench to Clinical Practice

**DOI:** 10.3390/jcm11072055

**Published:** 2022-04-06

**Authors:** Erica Rocco, Maria Chiara Grimaldi, Alessandro Maino, Luigi Cappannoli, Daniela Pedicino, Giovanna Liuzzo, Luigi Marzio Biasucci

**Affiliations:** 1 Department of Medical-Surgical Sciences and Biotechnologies, Cardiology Unit, ICOT Hospital, Sapienza University of Rome, 04110 Latina, Italy; ericarocco.md@gmail.com; 2 Department of Cardiovascular and Pneumological Sciences, Catholic University of the Sacred Heart, 00168 Rome, Italy; alessandromaino93@gmail.com (A.M.); luigi.cappannoli@gmail.com (L.C.); daniela.pedicino@policlinicogemelli.it (D.P.); giovanna.liuzzo@unicatt.it (G.L.); luigimarzio.biasucci@unicatt.it (L.M.B.); 3 Department of Cardiovascular Sciences, Fondazione Policlinico Universitario A. Gemelli IRCCS, 00168 Rome, Italy

**Keywords:** coronary microvascular dysfunction, endothelial dysfunction, biomarkers

## Abstract

Coronary microvascular dysfunction (CMD) is related to a broad variety of clinical scenarios in which cardiac microvasculature is morphologically and functionally affected, and it is associated with impaired responses to vasoactive stimuli. Although the prevalence of CMD involves about half of all patients with chronic coronary syndromes and more than 20% of those with acute coronary syndrome, the diagnosis of CMD is often missed, leading to the underestimation of its clinical importance. The established and validated techniques for the measurement of coronary microvascular function are invasive and expensive. An ideal method to assess endothelial dysfunction should be accurate, non-invasive, cost-effective and accessible. There are varieties of biomarkers available, potentially involved in microvascular disease, but none have been extensively validated in this heterogeneous clinical population. The investigation of potential biomarkers linked to microvascular dysfunction might improve the assessment of the diagnosis, risk stratification, disease progression and therapy response. This review article offers an update about traditional and novel potential biomarkers linked to CMD.

## 1. Definition and Pathophysiology

Coronary microcirculation is constituted by the vascular compartment of vessels with less than 500 μm diameter (pre-arterioles, arterioles and capillaries), which has a key role in the physiological modulation of cardiac perfusion. The endothelial monolayer can administer the exchange of fluids and metabolites and, furthermore, can manage vascular hemostasis. In case of a raised myocardial metabolic request, the coronary microvasculature modulates the peripheral vascular resistance and administrates the blood flow distribution that can reach a fivefold increase in healthy subjects [1].

Coronary microvascular dysfunction (CMD) relates to a broad range of clinical settings in which cardiac microvasculature is morphologically and functionally affected and it is associated with an impaired response to vasoactive stimuli [2].

Camici and Crea proposed a clinical–pathogenetic classification of CMD in four principal categories: (1) CMD in the absence of myocardial diseases and obstructive coronary artery disease (CAD), (2) CMD in myocardial diseases, (3) CMD in obstructive epicardial CAD and (4) iatrogenic CMD [2].

It is known that a wide spectrum of agents and cardiovascular risk factors such as chronic illness [3], diabetes, metabolic syndrome, smoking [4] and hemodynamic forces [5,6,7] can disturb the homeostasis of endothelial cells and thus determine CMD [8].

The endothelial dysfunction relies on four principal effectors: inflammation, platelet activation, hemodynamic forces and autonomic dysfunction.

Inflammation is inevitably connected with both microvascular endothelial dysfunction and atherosclerosis pathogenesis. CMD seems to be triggered by the low-grade inflammatory state such as epicardial coronary artery disease [9,10]. 

The endothelial cell activation, triggered by inflammation, increases the production of reactive oxygen species (ROS), enhances the expression of adhesion molecules promoting platelet and leukocyte adhesion and activation and leaks the endothelial barrier [11].

Platelet activation, microvascular thrombosis and distal embolization can affect endothelial microcirculation function, enhancing vasoconstriction and inflammation [12]. In this context, the interplay between platelet CD40L and endothelial CD40-receptor is a relevant trigger for inflammation and thrombosis [13].

Arterial hypertension can help elicit the atherosclerotic process both in epicardial arteries and in coronary microcirculation [14]. Shear stress and hemodynamic forces activate molecular pathways in the endothelium that can influence its structural and functional phenotype, resulting in microvascular dysfunction and injury [14]. 

The imbalance between the sympathetic and parasympathetic tone enhances vasoconstriction and endothelial damage of coronary microcirculation [15]. In general, adrenergic-derived vasoconstriction is relevant in clinical situations in which normal non-neural vasodilator mechanisms are impaired, such as dyslipidemia and diabetes, also involved in CMD pathogenesis [15].

## 2. The Current State of the Art for the Diagnosis of CMD

The gold standard for the diagnosis of CMD consists of invasive coronary functional tests [16].

First, a correct invasive assessment of coronary microcirculation is based on the examination of the endothelium-independent microvascular vasodilatation, estimated by both the coronary flow reserve (CFR) and the index of microvascular resistance (IMR). Second, the endothelium-dependent dysfunction can be evaluated by the response to intracoronary acetylcholine provocation test (ACh-test) [16,17,18,19,20,21]. 

The first mechanism is tested through intracoronary vasodilator injection (i.e., adenosine). CFR is measured as the ratio between the maximal coronary flow, after vasodilator-induced hyperemia, and the resting state. CFR reflects both epicardial and microvascular response [22].

IMR is the result of the multiplication of the distal coronary pressure with the mean transit time of saline flush at ambient temperature, in the context of adenosine-triggered hyperemia. IMR is specific for the assessment of the microcirculation, and it is not affected by resting hemodynamic state [23].

CMD is characterized by CFR values below 2.0–2.5 or elevated IMR, generally >25, and/or evident vasoconstriction in response to the ACh-test [16,17].

In addition, patients with CMD could exhibit a slow contrast flow on coronary angiogram, defined as the “coronary slow flow phenomenon”, because of an increased coronary microvascular resistance.

The Coronary Vasomotion Disorders International Study Group (COVADIS) established the international standardized diagnostic criteria of CMD based on clinical presentation, absence of obstructive epicardial coronary artery disease, evidence of myocardial ischemia through non-invasive testing and invasive assessment of impaired coronary microvascular function [16,17]. 

However, standard techniques to measure microvascular function (i.e., CFR, IMR and ACh-test) are invasive, laborious and costly [24]. Moreover, the functional assessment of CMD requires additional specific equipment and devices, not always available on routine coronary angiographies on a large scale.

Therefore, CMD is still an infrequent and often missed diagnosis, leading to the underestimation of its clinical importance. The prevalence of CMD seems to be on the rise, affecting about 50% of patients with chronic coronary syndromes and more than 20% of those with acute coronary syndrome (ACS) [1]. A post hoc analysis of a large cohort highlighted the unfavorable prognosis of patients with ACS without obstructive CAD: the incidence of adverse event rates at one-year follow-up was 15.5%, including 3.3% of death and acute myocardial infarction (AMI) [24].

In recent years, numerous non-invasive tests (e.g., transthoracic Doppler-echocardiography evaluating the coronary flow velocity reserve (CFVR), cardiac magnetic resonance (CMR), computed tomography coronary angiography (CTCA), positron emission tomography (PET)) have been indicated for the assessment of CMD [16]. However, most of them have some limitations (Table 1).

In the first instance, despite a high positive predictive value, the sensibility of these methods is partially hindered by the differential diagnosis of obstructive CAD that is mandatory to be ruled out with prior use of invasive coronary angiography or CTCA. Moreover, these non-invasive diagnostic tests contemplate the exclusive use of vasodilators (e.g., adenosine or dipyridamole), and can only establish the coronary vasodilator capacity, limiting the discrimination of all the different subtypes of CMD. 

In this complex clinical setting, the measurement of traditional and novel biomarkers linked to endothelial dysfunction could improve the assessment of risk stratification, diagnosis, disease progression and therapy response.

Indeed, genetic and epigenetic differences contribute to modulating the endothelial function both in healthy subjects and in patients with cardiovascular diseases. In this landscape, noncoding RNAs represent attractive new biomarkers for their potential applications in personalized medicine.

## 3. Traditional Biomarkers: Troponin and Natriuretic Peptides

Cardiac troponin (Tn) represents an already well-validated biomarker of heart damage, crucial for the diagnosis of myocardial infarction and injury, but also in many different conditions and diseases [25].

The role of Tn as a biomarker in CMD is, however, less established [26,27]. Several studies, therefore, tried to investigate the possible role of Tn in CMD.

Research by Takashio et al. on 58 heart failure (HF) patients revealed that troponin T (TnT) plasma levels were increased in cases of CMD compared to healthy controls [28].

Fujii and collaborators demonstrated that patients undergoing elective percutaneous coronary angioplasty (PTCA) had higher post-PTCA values of IMR when abnormal troponin I (TnI) levels were detected, therefore suggesting a significant microvascular dysfunction [29]. These findings were also corroborated by another study that, similarly, found an analogous correlation between post-PTCA IMR values and plasma creatine kinase MB (CK-MB) [30]. A different study conducted on 55 patients treated with PTCA highlighted the same association between post-PTCA CFR value and Tn levels [31].

Interestingly, a more recent study conducted on 19 patients with stable angina did not find any correlations between Tn levels and the invasive assessment of CFR and found an only poor correlation with IMR [32]. Lastly, a larger study by Taqueti et al. conducted on patients with suspected CAD reported that higher Tn values were predictive of reduced CFR compared to patients without Tn increase. The association of low CFR and high Tn levels was related to an increased incidence of major adverse cardiovascular events (MACEs) [33]. The non-unique results of these studies underline, once again, how the role of Tn is yet to be fully understood in predicting CMD. 

Natriuretic peptides (NPs) are well-known diagnostic and prognostic biomarkers of HF [34] and have proved to be useful in clinical decision making and risk stratification for hospital readmission of HF patients [35]. Indeed, only a few studies associate NPs and CMD.

The two primary NPs are the atrial natriuretic peptide (ANP), released when atrial wall stretching occurs, and the brain natriuretic peptide (BNP), secreted by ventricular myocytes in case of volume overload. Furthermore, the N-terminal prohormone of BNP (NT-proBNP) has an established role and clinical use as a biomarker. Both NPs control fluid homeostasis, natriuresis and express dose-dependent vasoactive effects.

ANP is essential for endothelial homeostasis through autocrine and paracrine secretion. In subjects with a high-salt diet, the vasoconstriction of the microvasculature of the skin in response to low-dose ANP infusion was observed, with decreased capillary density and increased renal vascular resistance [22]. When higher doses of ANP were administered, it conversely resulted in skin vessel dilatation and blood pressure reduction [36].

Moreover, patients presenting both CMD and left ventricle (LV) diastolic dysfunction showed increased levels of plasma NT-proBNP compared to healthy subjects [37].

In patients affected by symptomatic hypertrophic cardiomyopathy without CAD, Knaapen et al. observed a reduced myocardial blood flow reserve (MBFR) assessed by PET as an index of microvascular dysfunction. In these patients, NT-proBNP was inversely correlated with MBFR [38]. Using a different technique, Mitchell et al. assessed the MBFR by CMR in patients without overt CAD and, once again, they found an inverse association between NT-proBNP levels and MBFR [39]. Taken together, high NT-proBNP plasma levels might be related to CMD.

## 4. Endothelial Microvascular Inflammation: Nitric Oxide (NO), Myeloperoxidase (MPO), Asymmetric Dimethylarginine (ADMA), Symmetric Dimethylarginine (SDMA), Calprotectin, C-Reactive Protein (CRP)

Inflammation is widely involved in the pathogenesis and progression of cardiovascular diseases [40,41,42,43,44,45] and it has also a pivotal role in CMD. Inflammation triggers endothelial dysfunction, platelet activation, vascular tone reactivity, atherosclerosis and plaque instability. The activation of both innate and adaptive immunity participates in the pathogenesis of coronary syndromes with the involvement of epicardial arteries, myocardium and microvasculature [41,42,43]. CMD itself induces the activation of molecular pathways converging to inflammation, atherosclerosis progression and myocardial fibrosis [46].

Growing evidence has highlighted CMD as having a pivotal role in HF with preserved ejection fraction (HFpEF) pathogenesis, corroborated by the elevation of circulating inflammatory biomarkers in this disease [47].

In experimental models of cardiovascular disease, diabetes and obesity, systemic inflammation affected endothelial nitric oxide (NO) homeostasis, compromising the cGMP and PKG molecular pathway [48]. Moreover, endothelial NO participates in the regulation of leukocyte adhesion to vascular cells, platelet function and microvascular tone. NO bioavailability can be impaired by both direct ROS-related inactivation and endogenous NO synthetase (NOS) inhibitors [49].

Local and systemic inflammation, enhancing the innate immunity, results in impaired NO production and amplified vascular adhesion molecules expression, therefore promoting the polymorphonuclear neutrophils (PMNs) adhesion to the endothelial layer. Once they are activated, PMNs release myeloperoxidase (MPO), an enzyme catalyzing the generation of ROS and nitrogen-derived reactive species (RNS), thus promoting oxidative damage [49].

MPO has proved to be responsible for the pathogenesis of atherosclerotic disease, ACS, HF and cardiovascular comorbidities [50] and it is a predictor of cardiotoxicity from cancer drugs [51]. MPO’s role in vascular dysfunction is due to the formation of hypochlorous acid in the subendothelial glycocalyx, leading to direct and indirect NO bioavailability reduction and, therefore, endothelial damage and dysfunction [52].

Asymmetric dimethylarginine (ADMA), an endothelial NOS inhibitor, is increased in subjects with endothelial dysfunction and has emerged as a potential predictor of cardiovascular diseases [53]. ADMA proved to be an independent risk factor for long-term adverse cardiovascular events [54,55].

Symmetric dimethylarginine (SDMA) is an alternative methylation product of arginine and a stereoisomer of ADMA, but it does not inhibit NOS activity, and its role in CMD and cardiovascular diseases is less established [56].

ADMA is metabolized by the enzyme dimethylarginine dimethylaminohydrolase (DDAH), whose activity is regulated by the cell’s oxidative status.

DDAH inactivation, in case of increased production of ROS and RNS in endothelial cells, results in increased ADMA concentrations, leading to increased leukocyte and PMNs activation, with subsequent degranulation and MPO release. MPO-induced oxidative stress decreases DDAH activity, leading to further ADMA accumulation and reduced NO production, creating a vicious cycle boosting endothelial dysfunction [50].

Wang et al. concluded that higher levels of ADMA and SDMA predicted prevalent CAD and long-term risks of MACEs [56].

Hage et al. conducted a study on the role of NO availability, MPO, ADMA and SDMA in the pathogenesis of CMD in HFpEF, finding higher concentrations of MPO, uric acid and calprotectin in these patients, reflecting high PMNs activation in this inflammatory setting [57].

Calprotectin binds the advanced glycation end-products (RAGE) receptor and the toll-like receptors 4 (TLR-4), enhancing the endotoxin-induced dysfunction of endothelial cells and cardiomyocytes.

In their study on HFpEF patients with CMD, Hage et al. [57] found correlations between (1) elevated calprotectin concentrations, NYHA class and hypertension; (2) NO availability and SDMA concentrations; and (3) arginine/ADMA ratio with structural remodeling. 

Lastly, it is well-validated that in both ACS and obstructive CAD, the increase in plasma concentration of C-reactive protein (CRP) and other inflammation-related markers is linked to a worse outcome. Conversely, CAD patients with normal values of inflammatory markers have a better clinical outcome [52].

CRP has already been proposed as a biomarker for CMD [58]. The Reynolds Risk Score also incorporated CRP together with the traditional risk factors for a 10-year risk estimation of MACEs [59].

In patients with established CMD, higher CRP levels were observed, such as in subjects suffering from chronic stable angina [60,61].

CRP levels are disturbed by many endothelial pathways, leading to a possible daily fluctuation and therefore making them hardly interpretable over extended periods. Therefore, the role of CRP as a biomarker of CMD and its mechanisms affecting endothelial homeostasis remain to be fully understood [62].

## 5. Cell Adhesion Molecules: ICAM-1, VCAM-1, E-Selectin

Inflammation causes endothelial damage and involves leukocyte activation, adhesion and transmigration through the endothelial layer [63].

The two principal classes of leukocyte adhesion molecules on endothelium are the vascular and intracellular adhesion molecules-1 of the immunoglobulin superfamily (VCAM-1 and ICAM-1) and E-selectin.

E-selectin facilitates the initial leukocyte–endothelial interaction and rolling. This binding leads to leukocyte activation, firm adhesion and transendothelial migration mediated by ICAM-1 and VCAM-1 interaction. Soluble forms of these molecules in plasma are regarded as surrogates of their cellular expression [64,65].

The association between endothelial dysfunction, leukocyte recruitment and adhesion molecules has already been demonstrated [66].

Tousoulis et al. found that patients with microvascular angina have both elevated ICAM-1 and VCAM-1 compared to the control group [67]. Lupatelli et al. reported an inverse correlation between vasodilatation of the brachial artery with ICAM-1 and VCAM-1 expression in healthy individuals [68]. Miwa et al. found a significant increase in E-selectin and ICAM-1 in patients affected by angina [69].

Vaccarino et al. observed the elevation of ICAM-1 in CMD patients, without any significant differences in VCAM-1 levels [70]. Nevertheless, Siminiak et al. did not find any differences in plasma levels of adhesion molecules in patients with CMD compared to healthy controls [71].

## 6. Neuregulin-1

Neuregulin-1 (NRG1) is a component of the epidermal growth factor family. It is released from the microvasculature endothelial cells in several tissues, mainly the nervous system and the heart, in response to inflammation, ischemia and oxidative stress [72,73,74,75]. NRG1 has different isoforms and NRG1-β is the most investigated one [76,77]. NRG1 has a pivotal role in both the development and maintenance of the cardiovascular system [78].

NRG1 acts by binding tyrosine kinase transmembrane receptors (ErbB2, ErbB3 and ErbB4) and activating the PI3K and MAPK pathways in cardiomyocytes, thereby inhibiting apoptosis and inducing cardiomyocyte proliferation. Recent research confirms that NRG1 promotes angiogenesis [79,80] and that NRG1 itself is upregulated by hypoxia [81].

Like BNP, NRG1 has been reported to have a cardioprotective function and to participate in the adaptive response to HF.

In HF, both the ErbB2 and ErbB4 receptors result downregulated, whereas NRG1 and NRG1-β expression is upregulated proportionally to HF severity and mortality [82,83,84]. Nevertheless, NRG1 decreases as end-stage HF occurs [85].

In HFpEF, the implications of NRG 1-β may differ according to the ischemic or non-ischemic HF etiology [83].

Hage et al. [86] compared circulating NRG 1-β in 86 stable patients with HFpEF, in 86 patients with HF with reduced ejection function (HFrEF) and in 21 controls. In the HFpEF group, the median NRG1-β levels were higher than in the HFrEF group and lower than in the control group. 

The authors hypothesized a compensatory increase in NRG1-β from the microvascular endothelium, with a cardioprotective insight in non-ischemic HFpEF. This compensatory mechanism in response to oxidative stress might be overwhelmed in ischemic HFpEF and HFrEF. 

As a support to this hypothesis, the NRG1 pathway was upregulated in the setting of ischemia/reperfusion injury [87].

NRG1-β levels initially increase in patients with ischemia, but as HFrEF or HFpEF proceeds, the endothelium is unable to release supplementary NRG1-β and its concentrations decline. Indeed, NRG1-β signaling is downregulated in hypoxic versus normoxic areas [88] and NRG1 levels are inversely correlated with coronary stenosis [89].

Even though microvascular inflammation and CMD may occur in both HFrEF and HFpEF, it is suggested to drive the pathophysiology of non-ischemic HFpEF and it is associated with an increased risk of hospitalization and cardiovascular events [90].

Supporting this hypothesis, Shah et al. showed that CMD was present in 75% of patients with HFpEF and correlated with peripheral endothelial dysfunction [91]. Therefore, in non-ischemic HFpEF, a deteriorated concentration of NRG1-β, may reflect the attempt of the microvascular endothelium to respond against mechanical stretch, oxidative stress, inflammation and hypoxia [73,91].

In this milieu, NRG1-ErbB4 activation may act on both dysfunctional cardiomyocytes and endothelial cells, reducing the pro-inflammatory and pro-fibrotic signaling, thus improving ventricular and endothelial stiffness [92,93,94].

## 7. Renalase

Renalase is a flavin adenine dinucleotide-dependent amine oxidase, and it is primarily secreted by the renal proximal tubules and cardiomyocytes [95,96,97,98]. Flavoproteins catalyze the oxidation/reduction processes in metabolic pathways and play key roles in the biosynthesis of essential cofactors and hormones [99].

Despite this, a recent understanding of the intracellular role of renalase revealed that it might function as a cytokine, independently of its enzymatic properties, both in physiological and pathological states. Indeed, renalase secretion increases in response to catecholamine and ischemia triggers and impedes oxidative stress, thereby attenuating cardiac remodeling and fibrosis [100,101,102]. Moreover, renalase also showed an anti-inflammatory and anti-apoptotic role [103].

Renalase is linked to CMD-related risk factors, such as hypertension, insulin resistance and diabetes [95,104].

Given the role of inflammation in CMD pathogenesis [63], Safdar et al. [105] assessed the relationship between renalase, inflammatory markers and acute chest pain in 80 patients admitted at the emergency department and ruled out for AMI. Based on PET/CTCA results, patients were categorized as normal, CAD/calcification and CMD, according to their coronary phenotype. Median renalase values were higher in patients with CMD compared to patients with normal flows or CAD/calcification.

Renalase resulted as an independent predictor of CMD after adjustment for smoking, family history, obesity and Framingham risk score.

According to Safdar et al. [105], renalase resulted as an easily obtainable blood biomarker for CMD diagnosis in patients with chest pain, previously ruled out for AMI using contemporary assays, who may be referred for further invasive functional tests instead of being discharged at home.

In a similar study, Medvedev et al. [106] detected a different isoform of renalase in healthy individuals. They reported that CMD might be associated with different renalase isoforms, suggesting that both qualitative and quantitative evaluation might help to establish the role of renalase as a novel biomarker.

## 8. Serotonin

Serotonin or 5-hydroxytryptamine (5-HT) is mostly produced by serotonergic neurons in the central nervous system and enterochromaffin cells in the gastrointestinal tract and then collected by platelets from the bloodstream.

Serotonin released from activated platelets has a potent bidirectional role, causing both platelet aggregation and blood flow reduction through vasodilation via 5-HT1 receptors on endothelial cells and vasoconstriction via 5-HT2 receptors on vascular smooth muscle cells [107].

Increased serotonin levels in patients with CMD could trigger microvascular constriction via 5-HT2 receptors, with minimal effects on epicardial coronary arteries, and sensitize microcirculation to other vasoconstrictor agents [108,109,110].

Furthermore, Nemecek GM et al. [111] demonstrated that 5-HT stimulates the proliferation and migration of vascular smooth muscle cells and promotes atherosclerosis of epicardial arteries. Indeed, the plasma concentration of 5-HT was significantly higher in patients with CAD or vasospastic angina (VSA) than in those without it [112,113].

Recently, Odaka et al. [114] investigated the relationship between serotonin and CMD in 198 patients with angina without obstructive CAD. This study considered four clinical groups of patients: chest pain syndrome, CMD, VSA with CMD and VSA without CMD.

Serotonin plasma levels were significantly higher in patients with CMD compared to those without it. Furthermore, serotonin levels were similar in both VSA without CMD and chest pain syndrome groups.

This evidence highlights that plasma serotonin levels are linked to an atypical reactivity of the coronary microcirculation in comparison to epicardial coronary arteries in the CMD setting.

Authors found that the plasma serotonin level was the strongest predictor of CMD, identifying a potential diagnostic biomarker. Nevertheless, as coronary organic stenosis may trigger the elevation of serotonin concentrations, the benefit of serotonin as a biomarker for CMD might be confined in the setting of angina without obstructive CAD [112].

The role of selective serotonin reuptake inhibitors (SSRIs) in the cardiovascular system is a matter of interest and debate, and new evidence about their effect on CMD is needed. Depressive disorders are associated with elevated levels of inflammatory markers [115] that lead to endothelial dysfunction, increased expression of adhesion molecules and platelet activation [116,117].

SSRIs exhibit endothelium protective properties, reducing inflammation and improving calcium-/nitric oxide-mediated vasodilatation [118,119].

Pizzi et al. [120] demonstrated that sertraline therapy significantly reduced CRP and IL-6 levels and improved the endothelium-mediated vasodilatation compared to the placebo group. Moreover, Lekakis et al. [121] assessed the cardioprotective effects of SSRIs, such as reducing expression of VCAM-1 and ICAM-1 in aorta endothelial cells and adhesiveness to monocytes induced by TNF.

The potential CMD biomarkers previously analyzed are summarized in Supplementary Table S1.

## 9. Noncoding RNAs End Extracellular Vessels

Cardiac microvascular endothelial cells (CMECs) are actively involved in cardiac angiogenesis and homeostasis through exerting effects on metabolism and coronary blood flow regulation. CMECs dysfunction contributes to ischemic injury [122,123].

Previous evidence suggests that CMD can be considered as the pathological basis and the earliest stage of coronary heart disease and atherosclerosis [124,125].

The noncoding RNA landscape in cardiovascular research added novel insight into the pathophysiology of diseases. Among them, long noncoding RNAs (lncRNAs) and microRNAs (miRNAs) are the most characterized noncoding RNA molecules. In the era of a precision medicine approach to diseases, noncoding RNA may represent a new frontier as biomarkers [50,126]. 

LncRNAs lack clear protein-coding potential but are crucial for the modulation of gene expression and the pathogenesis of various cardiovascular disorders such as atherosclerosis, hypertension, diabetes, HF and ischemic heart conditions [127,128].

Although different lncRNAs have been related to the atherosclerotic process and endothelial function, there is still a lack of evidence about lncRNA and CMD in a human model.

LncRNA metastasis-associated lung adenocarcinoma transcript 1 (MALAT1), first recognized as a cancer promotor, is an endothelial cell-enriched lncRNA regulating cell migration and angiogenesis. MALAT1 engages in multiple cardiac diseases and different studies demonstrated that it is linked to endothelial cell regeneration [129]. It is one of the foremost promising lncRNAs involved in the angiogenic process of atherosclerosis, diabetic vasculopathy and retinopathy, and central nervous system injury. 

Different molecular mechanisms contribute to the pathogenesis of ischemia, such as oxidative stress, inflammation and the perturbation of mitochondrial function. Mitochondria act as arbiters of cell survival, thus enhancing endothelial cell repair or damage and ruling the apoptotic pathway. Additionally, mitochondrial dysfunction is strictly linked to endothelial dysfunction, and it triggers and aggravates many cardiovascular diseases [130,131].

Chen et al. demonstrated that MALAT1 modulates the microvascular function in a murine model of AMI, by affecting the microRNA 26b-5p/Mitofusin-1 (MFN1) signaling on the mitochondrial dynamics in cardiac endothelial cells.

MiR-26 has a validated anti-angiogenic role in endothelial cells; MFN1 promotes mitochondrial fusion, modulates angiogenic potential and protects cardiomyocytes against ischemia/reperfusion injury [132,133].

Silencing MALAT1 in CMECs perturbates mitochondrial function and enhances apoptotic pathways, thus aggravating endothelial dysfunction in acute and chronic settings.

LncRNA ST8SIA3, also called regulator of reprogramming (ROR), is a large intergenic noncoding RNA and participates in human microvascular endothelial cells (HMEC-1) homeostasis. ROR upregulates miR-26, NF-κB and JAK1/STAT3 pathways, involved in the atherosclerosis process. Qin et al. demonstrated that lncRNA ROR inhibited the growth and migration of HMEC-1 cells and therefore the capillary formation in vitro [134]. The silencing of ROR ameliorates atherosclerosis progression by the inhibition of miR-26, NF-κB and JAK1/STAT3 signaling.

LncRNA myocardial infarction-associated transcript (MIAT), previously related to ACS and CAD, is involved in diabetes-induced microvascular dysfunction [135]. Yan et al. demonstrated that MIAT participates in the pathogenesis of endothelial dysfunction in vitro and in a mouse retinal model [136].

MIAT acts as a sponge for miR-150-5p in endothelial cells, reducing miRNA availability for binding their target. VEGF, a key factor involved in angiogenesis, is one of the putative targets of miR-150-5p, potentially involved in MIAT-VEGF crosstalk [137]. MIAT knockdown significantly reduces the proliferation of endothelial cells in vitro, perturbs specific pathways involved in cell proliferation, migration and survival, thus alleviating microvascular dysfunction.

Different studies demonstrated that miR-126 is one of the most expressed miRNAs in endothelial cells of the heart and lungs and its reduced expression is associated with a loss of vascular integrity [138]. MiR-126 modulates endothelial cell proliferation and migration, promotes vascular remodeling and prevents fibrosis in cardiovascular pathophysiology [139].

MiR-126 safeguards the endothelium by downregulating the PI3K/AKT/NOS signaling pathway, involved in cell survival, vascular inflammation and ischemia/reperfusion-induced injury [140,141]. In addition, miR-126-5p has been reported as a relevant biomarker for the severity of CAD in stable patients [142].

Zhang et al. demonstrated that lncRNA MALAT1 causes brain microvascular dysfunction by sponging miR-126 and regulating the PI3K/AKT pathway, thus enhancing apoptosis and endothelial dysfunction. As the overexpression of MALAT1 inhibits the proliferation and enhances the oxygen/glucose deprivation-induced apoptosis of the human brain microvascular endothelial cells, it might also provide a possible therapeutic target [143].

Microvascular endothelial cells can release heterogeneous classes of extracellular vesicles (EVs) into the bloodstream and extracellular matrix as apoptotic bodies, microparticles and exosomes.

These nano-sized endothelial EVs function as messengers for intercellular communication and participate in tissue homeostasis. EVs represent a signaling and delivery system for different classes of proteins, lipids and nucleic acids including noncoding RNAs to multiple types of cells. Indeed, they contribute to the pathogenesis of inflammation, apoptosis, angiogenesis, vascular tone control, endothelial damage and dysfunction. In a disease setting, they can act as both causes and consequences of the pathogenic processes, making them potentially promising biomarkers [144,145]. In recent years, different studies have shown that EVs carrying miRNAs are involved in the pathophysiology of the vascular endothelium [146].

Notably, the endothelial cell apoptosis triggers the packaging of miR-126-3p into apoptotic bodies, released to the extracellular space and to adjacent endothelial cells, exerting a protective effect. The paracrine transfer of miR-126-3p from apoptotic endothelial cells upregulates the chemokine CXCL12 within the recipient cells, supporting endothelial function and reducing atherosclerosis [147].

## 10. Conclusions and Future Perspectives

Although much remains to be elucidated about the molecular pathology, clinical assessment and treatment of CMD, an accurate diagnosis is crucial for those patients’ best outcome and prognosis.

Recent advances in diagnostics and technology added new tools for invasive measurements and non-invasive imaging for CMD, improving the clinical management. 

The use of biomarkers might not replace the intracoronary diagnostic workup, but it would help to identify a specific group of patients committed to additional diagnostic tests. This may lead to a proper diagnostic workup and targeted therapy with a consistent impact on hospitalizations and overall prognosis.

Still, none of the traditional and novel potential biomarkers have been extensively tested in this clinical population.

As CMD might be considered a systemic disease with multiple clinical phenotypes and evolution, different biomarkers could be mentioned. The wide range of potential biomarkers reflects the heterogeneity of this population of patients.

**Figure 1:**
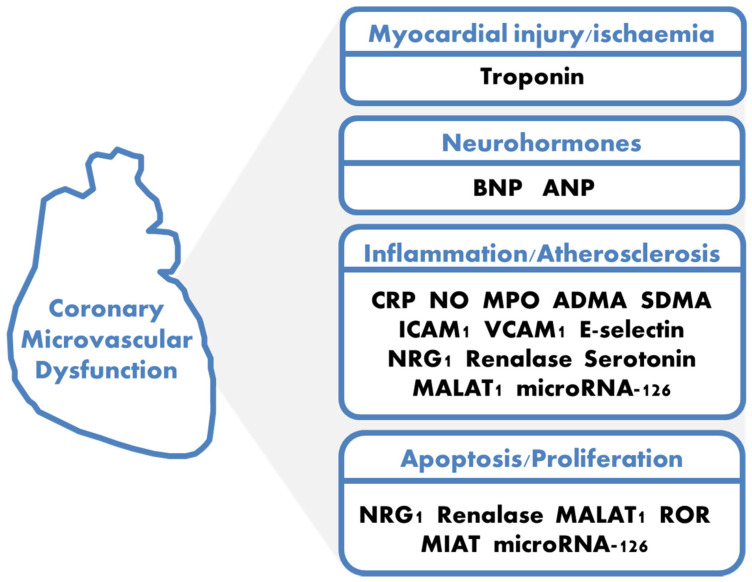
Coronary microvascular dysfunction traditional and novel potential biomarkers. ADMA = asymmetric dimethylarginine; ANP = atrial natriuretic peptide; BNP = brain natriuretic peptide; CRP = C-reactive protein; ICAM-1 = intracellular adhesion molecules-1; MALAT1 = metastasis-associate lung adenocarcinoma transcript 1; MIAT = myocardial infarction–associated transcript; MPO = myeloperoxidase; NO = nitric oxide; NRG1 = neuregulin-1; ROR = regulator of reprogramming; SDMA = symmetric dimethylarginine; VCAM-1 = vascular adhesion molecules-1.

Among all the potential biomarkers related to CMD, this review reported a selection of them involved in inflammation, apoptosis, atherosclerosis and fibrosis according to their translational implications (Figure 1).

One of the emerging fields of application of new potential biomarkers of microvascular dysfunction might be the HFpEF [148], as it shares with CMD the same inflammatory pathophysiological background. Recent evidence points at the comorbidity-associated systemic inflammation as a driving factor of HFpEF, inducing CMD and activating different molecular pathways converging on myocardial fibrosis [149]. On this basis, a deep knowledge of CMD and HFpEF may open new frontiers in improving diagnosis and targeted therapies. 

Further research is needed to assess the clinical relevance of existing candidate biomarkers and to identify new ones.

## 11. Limitations and Methodology

This review reported a selection among all the emerging potential biomarkers related to CMD, chosen according to their translational perspectives. A critical appraisal of the collected studies was conducted to carry out a reference list. The electronic research identified significant basic science and clinical research articles, outstanding papers and reviews, published between 1986 and 2022 and collected in the main datasets (PubMed, Science Direct, Scopus, Excerpta Medica Database and Cochrane). The articles were screened to identify their relevance according to the following items in the title, abstract and keywords: (“coronary microvascular dysfunction” OR “microvascular dysfunction”) AND (“endothelial dysfunction” OR “endothelial inflammation” OR “coronary endothelial dysfunction” OR “coronary endothelial inflammation”). The bibliographies of all identified papers were then examined for further relevant literature.

## Figures and Tables

**Table 1:** Characteristics of non-invasive methods for coronary microvascular dysfunction assessment.

Modality	Agent	Pros	Cons
Transthoracic Doppler echocardiography	Adenosine/Dipyridamole	Easily accessible. No radiation exposure.	Need previous rule-out of obstructive CAD. Operator-dependent.
Myocardial contrast echocardiography	Echocardiographic contrast substance	No radiation exposure. Assessment of global perfusion.	Lacking availability of standardized commercial software. Operator-dependent.
Positron emission tomography (PET)	Adenosine tracer (15O-H_2_O, 13 Nammonia, 82 R(b)	Reference standard of non-invasive methods. Assessment of global perfusion at the same time.	Difficult availability. Expensive. Radiation exposure. Need previous rule-out of obstructive CAD.
Cardiac Magnetic Resonance (CMR)	Adenosine/Regadenoson Gadolinium-based substances	No radiation exposure. Assessment of global perfusion. Used in the setting of obstructive CAD and structural heart disease.	Difficult availability. Expensive. Nonlinear relationship of tissue contrast concentration and MR signal intensity. Need of specific protocol.
Computed Tomography (CT)	Adenosine/Regadenoson Iodine-based contrast agent	Assessment of global perfusion at the same time. Used in the setting of obstructive CAD.	Need of further validation. Radiation exposure.

## Supplementary Materials

The following are available online at https://www.mdpi.com/article/10.3390/jcm11072055/s1, Table S1: Biomarkers in coronary microvascular dysfunction.
