# Supplementary material for: Advances and Challenges in Biomarkers Use for Coronary Microvascular Dysfunction: From Bench to Clinical Practice
Source: J Clin Med. 2022 Apr 6;11(7):2055. doi: 10.3390/jcm11072055 (PMC8999821; doi:10.3390/jcm11072055)
Supplement: Supplementary file 1 [file jcm-11-02055-s001.zip › jcm-1628092-supplementary.pdf]

Supplementary Table S1. Biomarkers in coronary microvascular dysfunction.

Candidate biomarker	Biological class	Site of production	Cardiovascular function	Clinical implication and use	References
Cardiac troponins (T and I)	Cardiac enzymes	Cardiomyocytes	To hold the actin-tropomyosin complex in place	Main diagnostic biomarker of myocardial infarction and injury.<br>Uncertain correlation with IMR, CFR and CMD.	[25-33]
Natriuretic peptides (ANP and BNP)	Protein neuro-hormones	Ventricular ed atrial cardiomyocytes	Vascular tone regulation, diuresis and natriuresis, antagonism of the renin-angiotensin system	Diagnostic and prognostic marker of HF.<br>Uncertain correlation with CMD and myocardial BFR.	[34-39]
Myeloperoxidase (MPO)	Protein enzyme	Polymorphonuclear neutrophils (PMNs)	Generation of oxygen- and nitrogen-derived reactive species promoting oxidative damage of pathogens	Indirect reduction of nitric oxide (NO) bioavailability leading to CMD.<br>Predictor of cardiotoxicity from cancer drugs.	[49-57]
Asymmetric dimethylarginine (ADMA)	Methylation product of amino acid arginine	Probably intracellular, ubiquitously, with extracellular deposition mainly in kidney and liver	Endogenous inhibitor of endothelial nitric oxide synthase (eNOS), reducing NO bioavailability<br>Increased leukocyte and PMN activation	Independent risk factor for long-term adverse cardiovascular events.<br>Indirect reduction of NO bioavailability leading to CMD.	[53-57]
Symmetric dimethylarginine (SDMA)	Alternative methylation product of amino acid arginine (enantiomer of ADMA)	Probably intracellular, ubiquitously, with extracellular deposition mainly in kidney and liver	Still not clear<br>It lacks eNOS inhibitory activity	Higher levels of predict coronary artery disease and long-term MACEs.<br>Correlation with diastolic dysfunction.	[53-57]
C-reactive protein (CRP)	Annular pentameric protein	Liver and adipose tissue	Acute-phase protein that binds to lysophosphatidylcholine on the surface of dead or dying cells to activate the complement system via C1q	Independent predictor of MACEs in CAD.<br>Uncertain role in predicting CMD.	[40, 58-62]
ICAM-1<br>VCAM-1	Transmembrane glycoprotein receptor	Ubiquitously in vascular endothelial cells	Leukocyte–endothelial interaction and rolling	Positive correlation with CMD.	[63-71]

E- selectin	Transmembrane glycoprotein receptor	Ubiquitously in vascular endothelial cells	Leukocyte activation, firm adhesion and transendothelial migration	Positive correlation with CMD	[64-65]
Neuregulin-1 (NRG1)	Peptide of the epidermal growth factor (EGFR) family	Vascular endothelial cells, mainly of the nervous and cardiovascular systems	Development and maintenance of the cardiovascular system	Expression upregulated with progression of heart failure (HF)<br>Decreased levels in end-staged HF	[72-94]
Renalase	Flavin adenine dinucleotide-dependent amine oxidase	Renal proximal tubules, cardiomyocytes, liver, pancreas, skeletal muscle and reproductive system	Anti-oxidative, anti-inflammatory, anti-apoptotic role	Positive correlation with CMD	[95-106]
Serotonin (5-HT)	Product of amino acid tryptophan	Serotonergic neurons of central nervous system and enterochromaffin cells of the gastrointestinal tract	Platelet aggregation, vasodilation and vasoconstriction, proliferation and migration of vascular smooth muscle cells, atherogenic role.	Positive correlation with CMD	[107-121]
